# Introduction of Curdlan Optimizes the Comprehensive Properties of Methyl Cellulose Films

**DOI:** 10.3390/foods12030547

**Published:** 2023-01-26

**Authors:** Liang Zhang, Liang Xu, Jin-Ke Ma, Yun-Yue Ye, Ying Chen, Jian-Ya Qian

**Affiliations:** School of Food Science and Engineering, Yangzhou University, Huayang Xilu 196, Yangzhou 225127, China

**Keywords:** methylcellulose, curdlan, edible film, blend ratio, microstructures, compatibility, physical properties

## Abstract

The good oxygen barrier and hydrophobic properties of curdlan (CL) film might be suitable complements for MC film, and its similar glucose unit and thermal-gel character might endow the methyl cellulose (MC)/CL blended system with compatibility and good comprehensive properties. Thus, MC/CL blended films were developed. The effects of MC/CL blend ratios on the microstructures and physical properties of the blends were characterized by using Fourier transform infrared spectroscopy (FTIR), X-ray diffraction (XRD), scanning electron microscopy (SEM), oxygen and water vapor permeability testing, dynamic mechanical analysis (DMA), light transmittance testing, tensile testing, hydrophilic property testing, and water solubility testing. The introduction of CL affected the molecular aggregation and crystallization of the MC molecules, suggesting MC–CL molecular interactions. The cross-sectional roughness of the MC/CL film increased with an increase in CL content, while the surface of the MC/CL 5:5 film was smoother than those of the MC/CL 7:3 and 3:7 films. Only one glass transition temperature, which was between that of the MC and CL films, was observed for the MC/CL 7:3 and MC/CL 5:5 films, indicating the good compatibility of the MC and CL molecules at these two blend ratios. The hydrophobicity and water insolubility increased with the CL content, which was due to the combined effects of more hydrophobic cavities in the CL triple-helix and increased surface roughness. Increased oxygen barrier properties with increasing CL content might be a combined effect of the increased hydrogen bonds and hydrophilic ektexines of the CL triple-helix. The elongations of the blended films were higher than those of the MC film, which might be related to its increased water content. The MC/CL 7:3 and MC/CL 5:5 films retained the good light transmittance and tensile strength of the MC film, which corresponded well to their good compatibility and might be due to the effects of the MC–CL molecular interactions and the relative smooth morphologies. MC/CL 5:5 showed improved water vapor barrier properties, which might be due to its smooth surface morphologies. This research offers new MC based films with improved properties and good compatibility, providing great potential for use as edible coatings, capsules, and packaging materials.

## 1. Introduction

Petroleum-based plastics are extensively used as packaging materials due to their good comprehensive properties. However, larger amounts of plastics used during the past century has caused serious environmental problems, since they are not biodegradable [1]. In order to solve these environmental problems, different biodegradable materials are being developed [1,2]. The production of edible packaging, as a kind of degradable wrapping, has increased significantly during recent years. As edible and bio-degradable materials, polysaccharide [3], protein [4], and their blends [5] are being studied extensively as edible films and have great potential to ease environmental problems.

Commonly, polysaccharides have relatively higher chemical and environmental stability than proteins. Cellulose is the richest polysaccharide in the world. Methylcellulose (MC), as an easily prepared cellulose derivative, is safe, non-toxic, edible, and has good film-forming properties. It is soluble in water at low temperatures and almost insoluble in anhydrous ethanol, ether, and acetone. It is a hot gel which can form a gel state at high temperatures and undergoes gel-sol transition when re-cooled to lower temperatures [6,7]. MC film is smooth, transparent, and exhibits good mechanical properties [8]. However, the high oxygen permeability and water sensitivity (solubility and hydrophilic properties) of MC hinders its widespread usage. Blending is an effective way to improve the properties of edible film made from a single component. Nanoparticles such as montmorillonite and ZnO were used to improve the oxygen and water barrier properties of MC and hydroxypropyl methylcellulose films [9,10]; however, these nanoparticles might affect the ductility of MC film, they are not edible, and they could not be used in edible films. Chitosan was added to MC film to improve its hydrophobic property [11]. Ascorbic acid, *Rheum ribes* L. extract, apple peel extract and other active substances were found to enhance the barrier, antibacterial, and antioxidant capacities of the film [12,13,14]. However, the barrier and hydrophobic properties of the MC film were rarely improved at the same time, and most times, the improvement of these properties was at the cost of some other properties due to the finitely compatible or incompatible issues. 

Curdlan (CL) is obtained from microorganisms [15]. It is formed by glucose through the β-1, 3-glycosidic bond; it is also a type of polysaccharide, with thermal-gel properties. CL is insoluble in water and many organic solvents, but is soluble in alkaline solutions with pH > 12 [16]. Due to its biodegradability, non-toxicity, and film forming properties, it has been used as a gelling agent and stabilizer in food and as an edible packaging material [17]. However, most previous studies have focused on the alkaline CL based films [16,18,19]; our previous research has shown that a neutral CL film can be prepared, and this kind of CL film showed good oxygen barrier and hydrophobic properties [20]. Thus, it was expected that CL could be a good complement for MC film; that is, the blending of CL was expected to improve the oxygen barrier and hydrophobic properties of MC film. Moreover, the similar glucose unit and thermal gel properties might endow the blend system with good compatibility and comprehensive properties. 

In this research, different contents of CL were blended with MC to prepare blend films. The microstructures (molecular interactions, crystalline structures, morphological structures), barrier properties (oxygen barrier property, water vapor permeability), water resisting property (water solubility, contact angle, water content), mechanical properties, and light transmittance property of all the MC/CL films were investigated. This research offers new films with much better comprehensive properties, showing great potential for use as edible coatings, capsules, and packaging materials. 

## 2. Materials and Methods

### 2.1. Materials

MC (methoxy content: 29%; viscosity (2% *w*/*w*): 14 mPa·s; pharmaceutical grade) was purchased from Nanjing Reagent Co., Ltd. (Nanjing, China); CL (CG-01; water content: 7.85%; food grade) extracted from microorganism was purchased from Jiangsu Yiming Biological Technology Co., Ltd (Suqian, China). Polyethylene glycol (PEG) 400 and other reagents of analytical purity were purchased from Sinopharm Chemical Reagent Co., Ltd. (Shanghai, China). 

### 2.2. Preparation of Solutions and Films by Casting

Hot water (85 °C) was added to a beaker contained MC powder to disperse the MC. Under slow stirring, the dispersion was cooled to room temperature to dissolve the MC. Deionized water was used to adjust the MC film-forming solution to the concentration of 1% (*w*/*w*). 

For CL suspension preparation, a method based on the research of Li et al. [21] was adopted, with slight modification. A total of 160 mL deionized water was added to a beaker containing 2.4 g CL to disperse the CL for 1 h. Then, NaOH (80 mL, 3 M) was added to the beaker to dissolve CL with slowly stirring for 5 h, and HCl (1 M) was adopted to neutralize the CL system (pH 7). The neutralized CL suspension was centrifuged at 6870× *g* for 20 min, and the supernatant was discarded. The obtained precipitated gel was mixed with deionized water for washing. After washing 3 times, deionized water was added to adjust the cleaned CL neutralized gel to a concentration of 1% (*w*/*w*). The CL film-forming suspension was prepared by homogenizing the above CL neutralized gel for 5 min (10 × 30 s) at 10,000 rpm.

For the purpose of preparing blended films with different blend ratios (10:0, 7:3, 5:5, 3:7, and 0:10), the above prepared MC solution (1% (*w*/*w*)) and CL suspension (1% (*w*/*w*)) were blended according to the corresponding proportions, then 10% (*w*/*w*_(MC+CL)_) PEG was added as the plasticizer, followed by stirring for 0.5 h and defoaming in a vacuum oven for 2 h. Next, 70 g of the above film-forming blends was poured into a polystyrene Petri dish with a diameter of 15 cm to be dried in an oven at 37°C for 12 h. The film was peeled and held at an RH of 59 ± 2%. MC/CL 10:0 was used to represent the MC film, MC/CL 0:10 was adopted to describe the CL film, and the blend films with various MC/CL ratios (7:3, 5:5, and 3:7) were recorded as MC/CL 7:3, MC/CL 5:5, and MC/CL 3:7, respectively.

### 2.3. Thickness Measurement

A micrometer caliper with an accuracy of 0.001 mm was used to determine the thickness of the MC/CL blend films. Seven repetitions were tested.

### 2.4. Structure Characterization

#### 2.4.1. X-ray Diffractometry (XRD)

Tests were conducted on all the films using a X-ray diffractometer (D8 Advance, Bruker AXS, Karlsruhe, Germany) under the following conditions: wavelength of 0.154 nm, voltage of 40 kV, pipe flow of 40 mA, scanning range of 3–40°, scanning speed of 3°/min.

#### 2.4.2. Scanning Electron Microscopy (SEM)

A scanning electron microscope (Geminisem300, Carl Zeiss Corp., Oberkochen, Germany) was used to observe the film morphologies. The samples were cut into 6 cm × 6 mm stripes. Liquid nitrogen was used to quench the stripes to obtain the cross-sections. The cross-sections and the surfaces were glued onto the conducting resin for gold spraying and observation.

#### 2.4.3. Fourier Transform Infrared (FTIR) Spectroscopy

An FTIR spectrometer (Cary 610/670, Varian Co., Palo Alto, CA, USA) was used to test the molecular interactions of the films. The FTIR spectrum was based on 32 scans, with a scanning range of 4000 cm^−1^–400 cm^−1^ and a resolution of 4 cm^−1^.

#### 2.4.4. Dynamic Mechanical Analysis (DMA) Determination

A dynamic mechanical analyzer (Q800, TA, New Castle, USA) was used to measure the films’ dynamic mechanical properties. The film strips of 30 mm × 2 mm were used for testing. The testing conditions are displayed below—heating rate: 2 °C/min; frequency: 1 Hz; amplitude: 5 pm; strain: 0.07%. The tests were carried out in the range of 30 °C to 200 °C.

### 2.5. Physical Properties Characterization

#### 2.5.1. Transmittance Determination

The film’s light transmittance was tested based on the method of Liu et al. [22]. The film strips of 10 mm × 40 mm were placed vertically in a quartz colorimetric dish. A 759S UV-Vis spectrophotometer (Lengguang Technology Co., Ltd., Shanghai, China) was used to test the films at full wavelengths, from 200 nm to 800 nm. The transmittance values at 500 nm were marked as T_500_. Three repetitions were performed for each sample.

#### 2.5.2. Contact Angle Determination

The contact angle of the sample was measured by using a contact angle goniometer (OCA15EC, Dataphysics, Filderstadt, Germany). The film sample (7 × 2 cm^2^) was pasted onto the slide and placed horizontally on the movable platform. The attached micro-syringe was used to drop ultra-pure water (5 μL) quickly onto the surface of the film. The contact angle variation curves, along with the placing time, were recorded. Three repetitions were performed for each sample.

#### 2.5.3. Mechanical Property Determination

In the tensile mode, the film stripes with a size of 60 mm × 6 mm were tested by using a STX 200 tensile tester (Yishite Corp., Xiamen, China). The weighing sensor of 1000 N was chosen for the test, with a clamping distance of 40 mm and a crosshead speed of 10 mm/min. The tensile strength and elongation at break were calculated, according to previous research [20]. Seven repetitions were performed for each sample.

#### 2.5.4. Oxygen Permeability (*OP*) Determination

A Basic 201 gas permeability tester (Labthink Instruments Co., Ltd., Jinan, China) was used to measure film’s oxygen permeability at a temperature of 23 °C and an RH of 50% [23]. The film sample was used to separate the gas permeation cell into two compartments (permeation area of 38.48 cm^2^). Prior to testing, the air in the two compartments was evacuated for 12 h. Then, the upstream compartment was padded with oxygen gas. The *OP* of the film was calculated as follows:

(1) $$OP=\frac{OTR\times x}{\Delta P}$$

where *OP* was a unit of cm^3^ mm m^−2^ day^−1^ atm^−1^, *OTR* represented the oxygen transmission rate with a unit of cm^3^ m^−2^ day^−1^, *x* represented the film thickness (mm), and Δ*P* represented the pressure difference between the two compartments (atm).

#### 2.5.5. Water Solubility Determination

The film samples were dried at 105 °C until they reached an invariable weight. The above dried samples were precisely weighed (marked as *M_i_*) and submerged into deionized water (50 mL) with sustained swirling for 1 h at 20 °C. The solution was discarded and the remaining film was dried at 105 °C to an invariable weight (marked as *M_f_*). Equation (2) was used to calculate water solubility. Three repetitions were performed for each sample.

(2) $$\mathrm{water}\;\mathrm{solubility}\;(\%)=\frac{{(M}_{i}-M_{f})}{M_{i}}\;\times \;100$$

#### 2.5.6. Moisture Content Measurement

The GB 5009.3-2016 method was adopted to determine the moisture content of the films. The films were dried in an oven at 105 °C until a constant weight was reached. 

#### 2.5.7. Water Vapor Permeability (WVP) Determination

The *WVP* (g mm^−1^·s^−1^·Pa^−1^) of the film was measured according to the method of Liu et al. [22], with some modifications. The film sample was sealed on a 50 mL centrifuge tube containing 40× *g* anhydrous silica gel. The centrifuge tube was then placed in an ambient chamber containing distilled water at room temperature. The weight change of the centrifuge tube was recorded at an interval of 24 h for 7 d. Water vapor permeability was calculated by Equation (3).

(3) $$WVP=\frac{W\times x}{t\times A\times \Delta P}$$

where *W* is the increasing weight (g), *x* is the film thickness (m), *t* is the testing time (s), *A* is the infiltration area (m^2^), and Δ*P* is the water vapor pressure difference between the two sides of the sample. Three repetitions were performed for each sample.

### 2.6. Statistical Analysis

SPSS 19 (IBM Software Inc., NY, USA) was used to analyze the data, which were displayed as means ± standard deviations (SD). One-or two-way analysis of variance (ANOVA), based on Duncan’s multiple comparison tests, were used to compare the means. Numbers with different letters indicate a significant difference between the corresponding samples (*p* < 0.05).

## 3. Results and Discussion

### 3.1. Molecular Interactions

#### 3.1.1. FTIR Analysis

**Figure 1:**
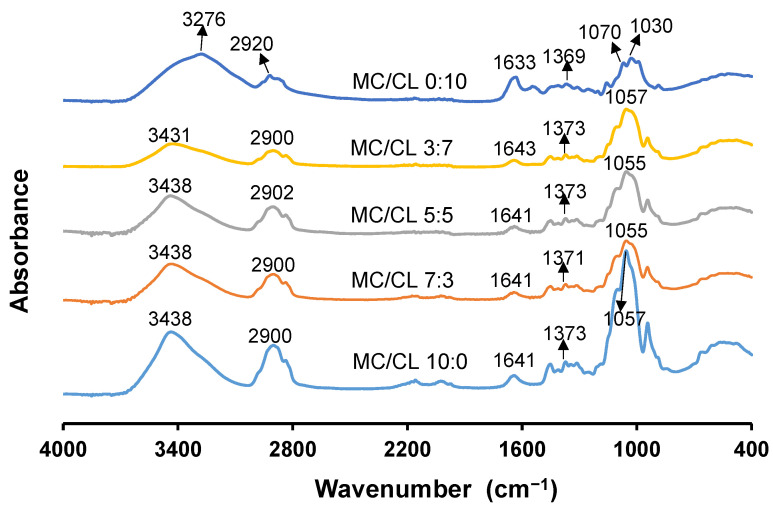
FTIR spectra of MC/CL films with different blend ratios.

The FTIR spectra of MC/CL films with different blend ratios are shown in Figure 1. For the MC film, the absorption peak of the C-O and C-O-C groups was observed at 1057 cm^−1^, the vibration peak of the C-H and CH_2_ groups was observed at 1373 cm^−1^, the vibration peak of water (H-O-H) was observed at 1641 cm^−1^, the absorption peak of the CH_3_ group was presented at 2900 cm^−1^, and the absorption peak of the O-H group was presented at 3438 cm^−1^. These corresponding absorption peaks and positions were similar with those reported for the hydroxypropyl methylcellulose film [24]. In the CL film, the C-O and C-O-C group absorption peak appeared at 1030 and 1070 cm^−1^, similar C-O and C-O-C group absorption peaks were observed for agar film [25] and starch film [26]; the absorption peak of the CH and CH_2_ groups appeared at 1369 cm^−1^, the water (H-O-H) group vibration peak appeared at 1633 cm^−1^, the absorption peak of the CH_3_ group could be observed at 2920 cm^−1^, and the stretching vibration peak of the O-H group appeared at 3276 cm^−1^. For the MC/CL 7:3, 5:5 and 3:7 films, the absorption peak of the C-O-C and C-O groups was presented at 1055, 1055 and 1057 cm^−1^, respectively; the absorption peak of the CH and CH_2_ groups was presented at 1371, 1373, and 1373 cm^−1^ respectively; The H-O-H group vibration peak was presented at 1641, 1641, and 1643 cm^−1^; the absorption peak of the CH_3_ group was presented at 2900, 2902, 2900 cm^−1^; and the stretching vibration peak of the -OH group appeared at 3438, 3438, and 3431 cm^−1^. Compared with the MC film and the blend films, the O-H group absorption peak of the MC/CL 3:7 film moved to a lower wavelength. Previous research has shown that the shift of the -OH group to the lower wavenumber indicated that more hydrogen bonds were formed [27,28,29]. Thus, the MC/CL 3:7 film had more hydrogen bonds than did the MC/CL 7:3 and 5:5 films. The increased hydrogen bonds might be contributed by the intramolecular and intermolecular interactions of CL-CL other than MC-CL molecular chains, since enhanced CL crystalline peaks (6° and 11°) were observed for the MC/CL 3:7 film in the following text (Section 3.2.1), along with the relative rougher morphological structures of the 3:7 film compared with those of the 7:3 and 5:5 films observed in the following part Section 3.2.2. The absorption wavenumber of the -OH group of other blended films was similar with that of the MC film. The absorption wavenumbers of other peaks of the blended films were also similar with that of the MC film.

#### 3.1.2. DMA

**Figure 2:**
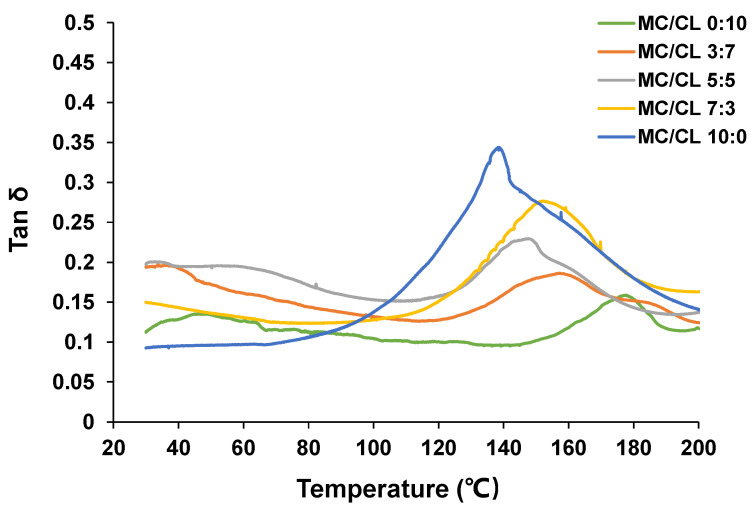
Tan δ curves of MC/CL films.

Figure 2 shows the tan δ curves of the MC/CL films. The peak temperature of this curve was recognized as the glass transition temperature. The glass transition temperature of the MC film occurred at about 138.9 °C, while the CL film showed the glass transition temperature at 177.6 °C, which was lower than that previously observed for the glycerol plasticized CL film [20]. This result might be because that PEG used as the plasticizer in this research had a much better plasticizing effect on the CL film. Similarly, previous research has shown that PEG had a better plasticizing effect on another kind of polysaccharide film—hydroxypropyl methylcellulose film [30]. The MC/CL 7:3 and 5:5 films presented only one peak, with the temperature of 152.2 °C and 147.6 °C, respectively, while the MC/CL 3:7 film presented two peaks at 156.9℃ and 184.5℃, respectively. Generally, blended films with only one glass transition temperature between that of the pure polymer-based films indicated good compatibility of the blended film [31]. For the immiscible/incompatible blends, original glass transitions of pure components were observed [32]. For a finitely or partially compatible blend, shifting or broadening of the glass transition regions in one or more components might be observed [32]. The only glass transition temperature for the MC film (138.9 °C) and CL film (177.6 °C) suggested that MC and CL molecular chains represented good compatibility at MC/CL blend ratios of 7:3 and 5:5. The two observed glass transition temperatures of the MC/CL 3:7 film shifted compared with that of the MC and CL film, which indicated that the system at this blend ratio had limited compatibility. These findings relating to compatibility corresponded well with those regarding the morphological structures observed in the following part Section 3.2.2.

### 3.2. Aggregated Structure

#### 3.2.1. Crystal Structure

**Figure 3:**
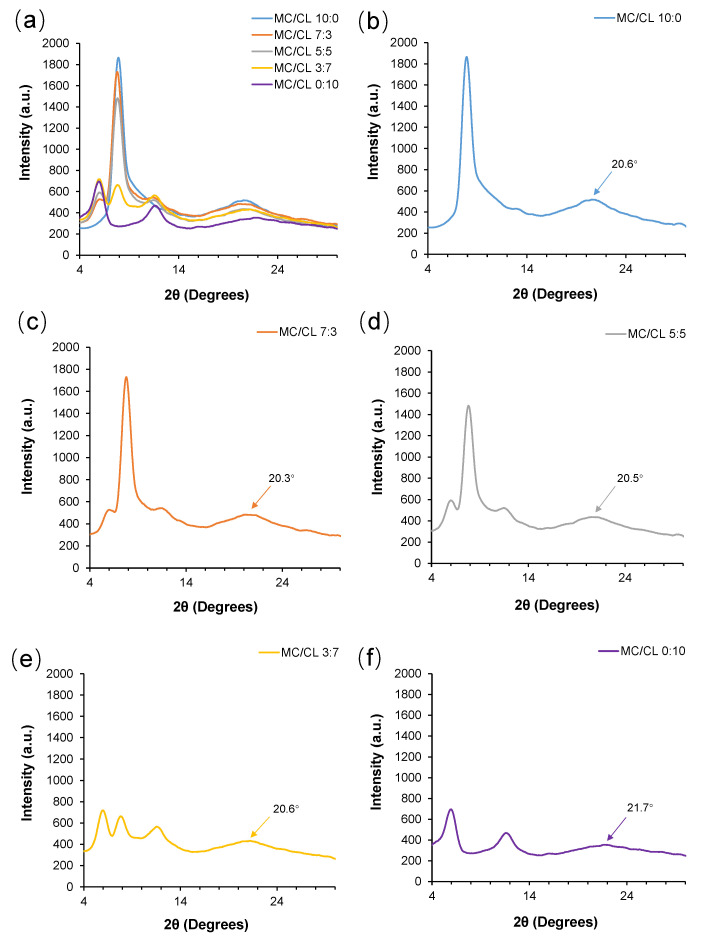
XRD spectra of MC/CL films with different blend ratios: (**a**) all the films displayed in one figure; (**b**) MC/CL 10:0; (**c**) MC/CL 7:3; (**d**) MC/CL 5:5; (**e**) MC/CL 3:7; (**f**) MC/CL 0:10.

Figure 3 shows the XRD spectra of MC/CL films with different blend ratios. The MC (Figure 3b) film showed a relatively sharp crystalline peak at about 7.9° and a wide amorphous peak at about 20.6°. Similar crystalline peaks were reported by Pinotti et al. [33] and Xiao et al. [34]. The sharp MC peak at about 8° corresponded to the trimethylglucose-type crystalline order in these films [35]. Two sharp crystalline peaks at 6.0° and 11.5°, and a wider amorphous peak at 21.7°, were observed for the CL film (Figure 3f). The CL crystalline peaks corresponded to the melting transition peaks observed by DSC [18]. A different crystalline type, which contained only one amorphous peak at 20.4°, was observed for the CL alkaline films [19]. This might be because there were more CL-CL hydrogen bonds for the CL neutralized film in this research, which could result in more ordered structures, while in the CL alkaline film, more CL-CL hydrogen bonds were destroyed by alkaline and more CL–water interactions were formed, which might hinder the CL chains from forming more ordered crystalline structures. All crystallization peaks corresponding to MC and CL appeared in the blended films (Figure 3c–e). The positions of the corresponding crystalline peaks did not change significantly, indicating that the crystalline types did not change after blending. The height and area of the crystalline peaks corresponding to one component (MC or CL) decreased with the increased content of the other component, indicating the decreased crystalline integrity and crystallinity of the corresponding crystals. These changes might be due to the introduction of CL, which might promote the MC-CL interactions and affect the MC molecular aggregation and crystallization. A similar trend of variation in the crystalline peaks with the blend component was observed for other blend systems, such as chitosan/MC films [33] and hydroxypropyl methylcellulose/hydroxypropyl starch films [30]. As shown with arrows in Figure 3, the position of the amorphous peak of the blended films varied little with the addition of CL, suggesting the MC-CL molecular entanglements in the amorphous region.

#### 3.2.2. Surface and Cross-Sectional Morphologies

**Figure 4:**
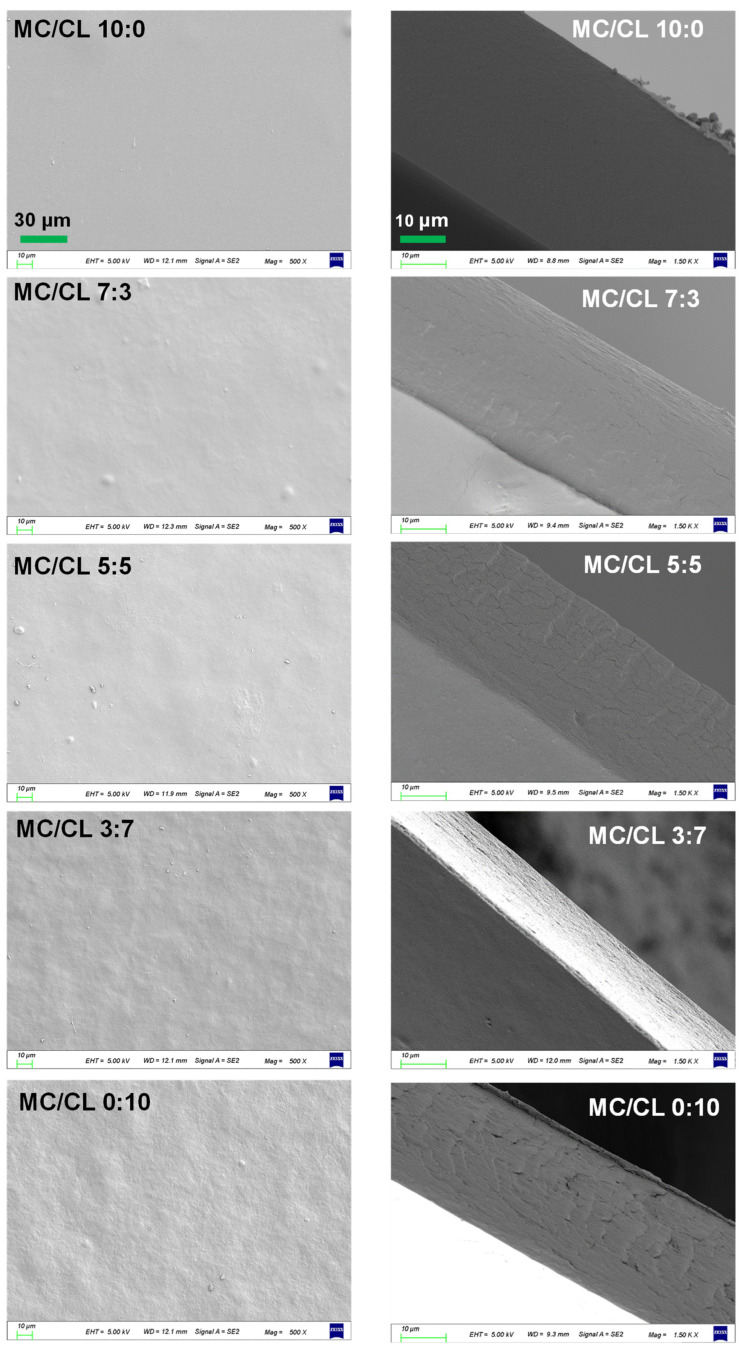
Surface and cross-sectional morphologies of the MC/CL films with different blend ratios. The same column uses the same scale bar.

Figure 4 shows the surface and cross-sectional morphologies of MC/CL films with different blend ratios. The MC film presented a clean and smooth surface, while the surface of the CL film was the roughest. The MC/CL 5:5 film showed the smoothest surface morphologies among all the blend films. The MC/CL 7:3 film presented relatively smooth surface morphologies, with only a few humps, while the MC/CL 3:7 film was much rougher, with many sags and crests. The MC/CL 7:3 and 5:5 films showed similar smooth surfaces to those of the chitosan/MC films, indicating the compatibility of the blend systems contributed by the interaction between the hydroxyl groups presented over the polymer matrices [11]. Similar compatibility could be inferred for the MC/CL 7:3 and 5:5 films compared with the chitosan/MC system. Increased surface smoothness was observed for the MC/CL film compared with the chitosan/CL film [36], indicating enhanced compatibility.

The cross-section of the MC film was very smooth, which was consistent with the image observed by da Silva et al. [8] and Khan et al. [37]. The cross-section of the CL film was rough, with some stripes, flaws, and holes, which was similar to the image observed by Zhou et al. [38]. The cross-sectional roughness increased with increasing CL content. The overall surfaces and cross-sectional morphologies of the blends were much smoother than those of the CL film, indicating that MC and CL showed certain compatibility. Moreover, all the MC/CL blends showed much smoother cross-sectional morphologies than those of the MC/chitosan films [33,39] and the hydroxypropyl methylcellulose/chitosan films [39], suggesting the much better compatibility of the MC/CL films.

### 3.3. Barrier Properties

#### 3.3.1. Oxygen Permeability Analysis

**Figure 5:**
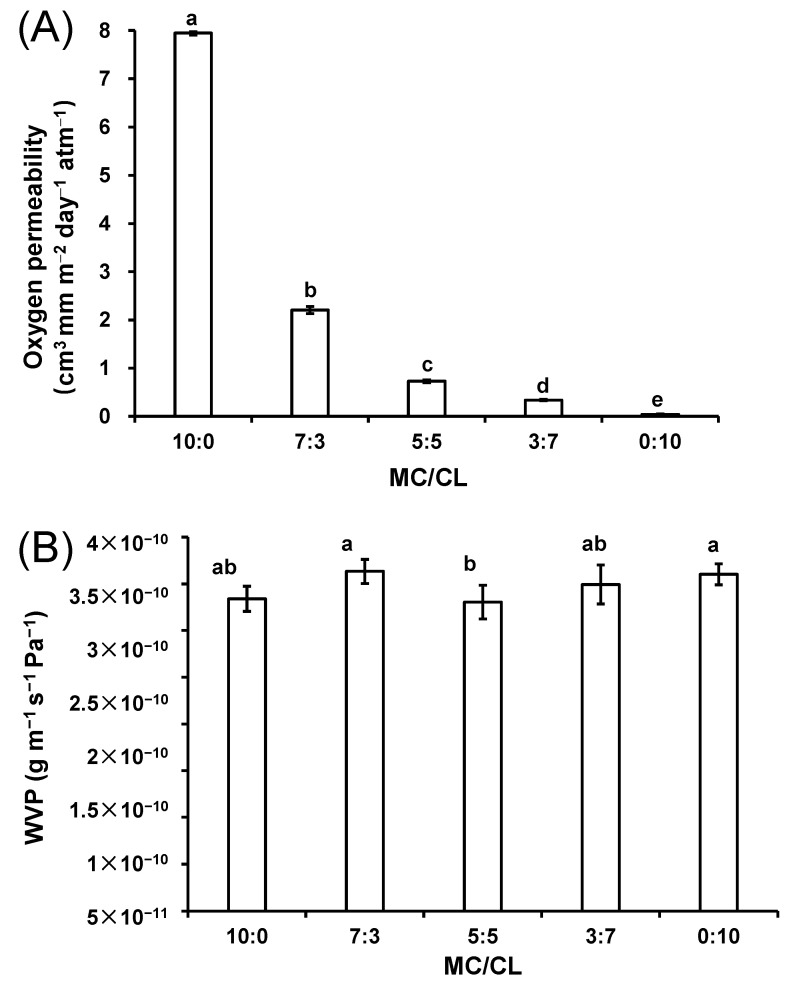
Oxygen permeability (**A**) and water vapor permeability (**B**) of the MC/CL films with different blend ratios. Different letters indicate significant differences (*p* < 0.05).

Oxygen permeability is an important index of edible film, exerting an important effect on its application in food preservation. Figure 5A shows the oxygen permeability of MC/CL films with different blend ratios. The oxygen permeability of the MC film was the highest. This might be because that the methoxy group on the film increased its interaction capacity with the oxygen molecules [34], while the oxygen permeability of the CL film was the lowest, as the many hydrogen bonds and the hydrophilic ektexines of the triple-helix might impede oxygen transmission. The oxygen permeability of the blended film decreased with the increase in CL content, suggesting that CL improved the oxygen obstructing performance of the MC film. The good oxygen obstructing property of edible film is much appreciated for packaging fruits and oils. A significant improvement in oxygen barrier properties on the MC films was observed in this research compared with those of the starch nanocrystal introduction method [34]. 

#### 3.3.2. Water Vapor Permeability

**Figure 6:**
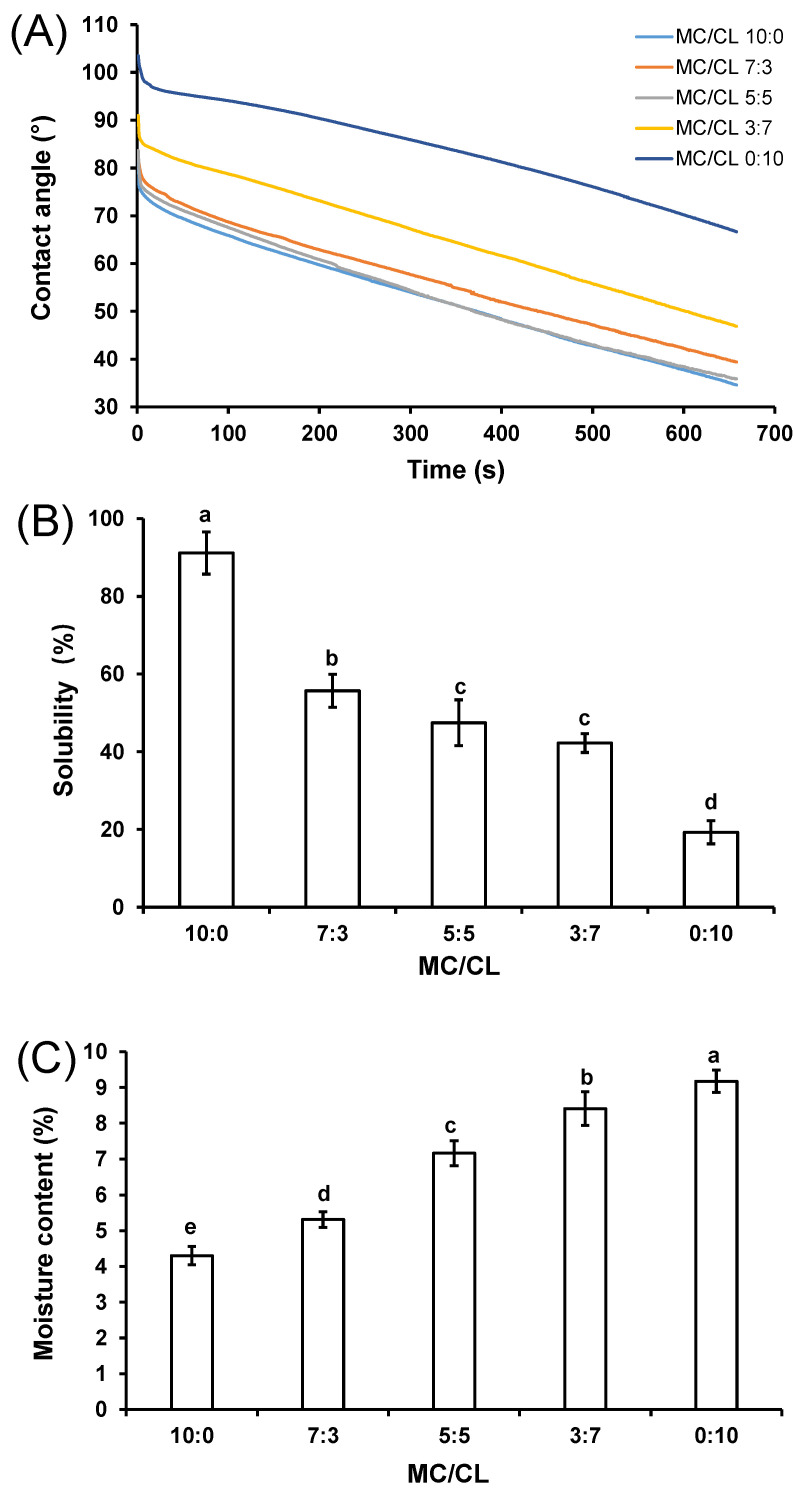
Contact angle (**A**), solubility (**B**), and water content (**C**) of the MC/CL films with different blend ratios. Different letters indicate significant differences (*p* < 0.05).

Figure 5B shows the water vapor transmittance of the MC/CL films with different blend ratios. The MC/CL 5:5 film had the lowest WVP value, and the other films had similar WVP values. The higher polarity of hydroxyls in MC and CL may facilitate the water molecules to condense, dissolving and permeating through the films. The condensation of water vapor and the dissolving step were preconditions for water vapor to permeate through the films [40]. CL showed the highest hydrophobicity (highest contact angle) (Figure 6A), which was expected to impede the water molecules from condensing and dissolving in the films, to some extent, decreasing the WVP. However, the obvious flaws and holes appearing in the cross-section and the higher water content (Figure 6C) of the CL film could facilitate water vapor molecules to diffuse across the film. Thus, these effects on the WVP of the CL film might balance out, resulting in a similar WVP for the CL and MC films. MC/CL 5:5 showed the lowest WVP, which might be due to its smoothest surface morphologies and highest compatibility among the blends. Decreased WVP is desired for retaining the stable property of foods under varied humidity.

### 3.4. Water Resisting Property

#### 3.4.1. Contact Angle

Figure 6A shows the water contact angles of the MC/CL films, with different blend ratios. The contact angle reflects the hydrophilicity/hydrophobicity of the film. The higher the water contact angle, the higher the hydrophobicity of the materials [11]. The contact angle of all the films decreased with the increase in time, which was because all the films can be wetted by water, and the wetting degree increased with the increase in time. The blended films containing no higher than 50% CL showed a similar contact angle with that of the MC film (MC/CL 10:0). Similarly, the gelatin/starch films containing no less than 50% gelatin had a similar water contact angle with that of the pure gelatin film [41]. The contact angle of the blended film was greatly determined by its continuous phase [41]. The MC/CL blend films containing no less than 50% CL showed the following order of contact angles: MC/CL 5:5 < MC/CL 3:7 < MC/CL 0:10. This might be because the films with higher CL content possessed more CL triple-helix structures, which showed more hydrophobic cavities [42], and the higher surface roughness of the films with higher CL content could also result in larger contact angle [18]. The contact angles of the MC/CL films were lower than those of the MC/chitosan films [11], indicating relatively lower hydrophobicity.

#### 3.4.2. Water Solubility Analysis

The water solubility of the MC/CL film is shown in Figure 6B. The water solubility of the MC film was about 90%, since MC contained a large number of hydroxyl groups. The water solubility of the CL film was about 20%, which might be due to the fact that CL contained triple-helix structures, with hydrophobic cavities inside [42]. The water solubility of the MC/CL film decreased significantly with the increase in CL content, which was due to the increasing content of the relative hydrophobic component—CL. The intermolecular interactions between MC and CL might also contribute to the decreased solubility of the blends through reducing the free hydrophilic sites [18,43,44]. Both increased water contact angle and decreased water solubility indicate the higher water-resisting property of the blended film, which is highly valued for packaging foods with relative higher water contents, such as meat and fish. 

#### 3.4.3. Water Content

Figure 6C shows the moisture content of the MC/CL films with different blend ratios. The moisture content of films increased with an increase in CL content, which was unexpected at first, since CL had the lowest water solubility (Figure 6B) and the highest water contact angle (Figure 6A). While the CL film was composed of the triple-helix structure of CL molecules, which contained the hydrophobic cavities and hydrophilic ektexines [42], the hydrophilic ektexines might endow its relatively higher hygroscopicity [20]. The MC film showed the lowest moisture content, which might be because the methoxy group substitution decreased the hydrophilic sites of the MC molecules and the MC-water molecular interactions. The increased moisture content of the MC/CL blends with an increase in CL content might be due to the decreased methoxy group of the MC component and the increased hydrophilic ektexines of the CL component.

### 3.5. Mechanical Property Analysis

**Figure 7:**
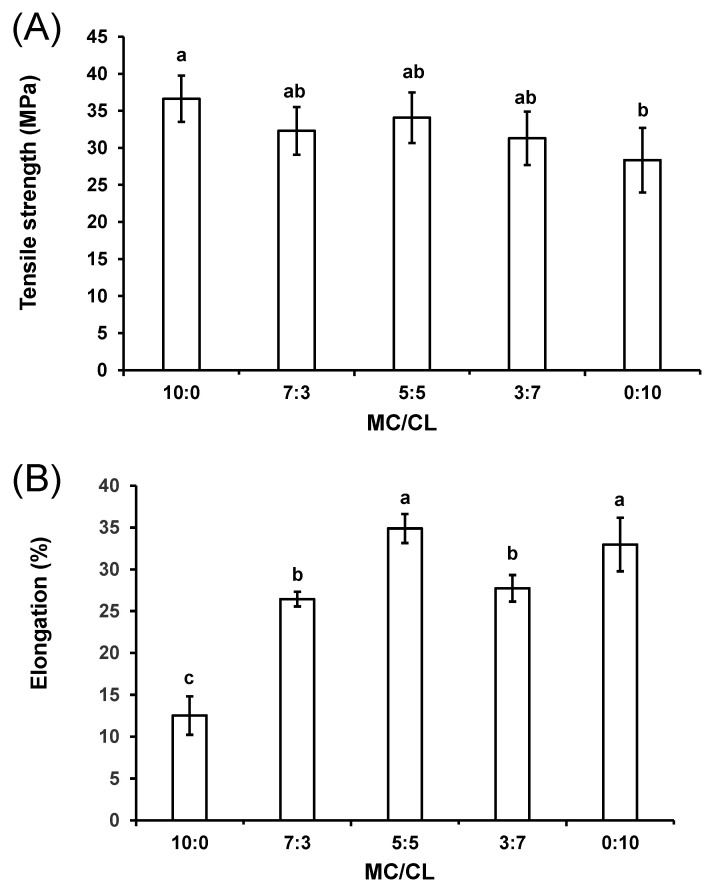
Mechanical properties of the MC/CL films with different blend ratios. Tensile strength (**A**); elongation (**B**). Different letters indicate significant differences (*p* < 0.05).

The tensile strength (A) and elongation (B) of the MC/CL films with different blend ratios are shown in Figure 7A and Figure 7B, respectively. The tensile strength of the MC film was 36.6 MPa, which was the highest among all the films, mainly being attributed to the smooth morphologies of this pure film [45]. The CL film showed a tensile strength of 28.3 MPa. The blended films showed a similar tensile strength value, which was not significantly different from that of the MC and CL films, indicating that the blended films showed a certain compatibility. The good tensile strength of the blended films might be due to the effects of the MC-CL molecular interactions inferred from the DMA and XRD curves and the smooth film morphology observed by SEM. 

The elongation at break of the MC/CL 7:3 and 3:7 films was higher than that of the MC film and inferior to that of the CL film, which might be related to the increased water content after the introduction of CL. The elongation of the MC/CL 5:5 film was higher than that of the MC film and similar to that of the CL film. The MC/CL 5:5 film showed the highest elongation among all the blends, which confirmed the former inference of its superior compatibility. This highest elongation might be mainly due to its smoothest surface morphologies among the blends. The elongation of the MC/CL film was much higher than that of the MC/chitosan films [11].

In terms of comprehensive mechanical properties, the MC/CL 5:5 film was the best among the MC/CL films, since it presented similar tensile strength to that of the MC film and similar elongation to that of the CL film, suggesting that it had the best flexibility (not only rigid, but also ductile). Flexibility is greatly valued for packaging, since it is connected to its load bearing and deformation degree.

### 3.6. Light Transmittance Analysis

**Figure 8:**
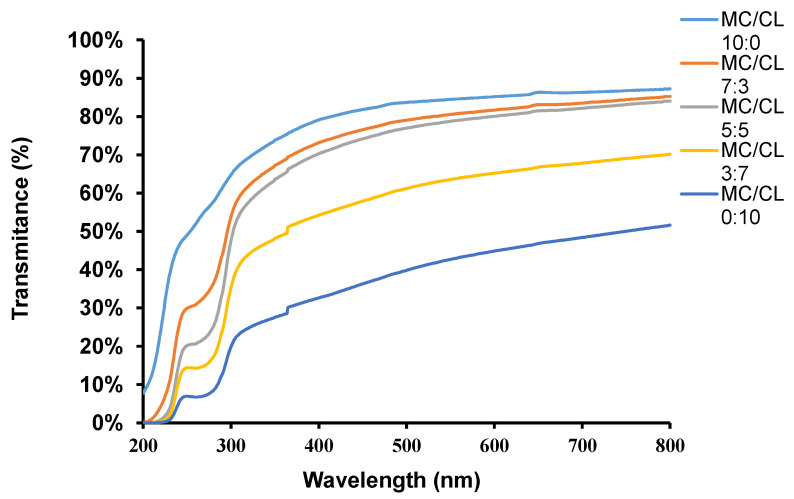
Light transmittance of the MC/CL films with different blend ratios.

The light transmittance variation, with wavelength and T_500_ of the MC/CL films with different blend ratios, are shown in Figure 8 and Table 1, respectively. The light transmittance of the MC film increased gradually with the increasing wavelength, and its transmittance was the largest among all the films at a fixed wavelength. The CL film showed the lowest light transmittance among all the films at a fixed wavelength. Fewer crystalline structures and more homogeneous matrices (smoother morphological structure, uniform phase, nonporous film) could increase the transmittance of the film [46,47], which was because these structures could decrease the difference in the refractive index. Thus, the highest light transmittance of the MC film might be due to its smoothest morphological structure, and the lowest light transmittance of CL might be due to its roughest morphological structures and small holes appearing on the cross-section. The light transmittance of the MC/CL 7:3, 5:5, and MC films was similar. When the CL content was higher than 50%, the light transmittances of the films decreased significantly with the increase in CL content. The low light transmittance of the 3:7 film confirmed the former inference of its lower compatibility. The changing trends of light transmittance with the CL blend ratio corresponded to the variation trends of the smoothness of their cross-sectional morphological structures. The light transmittances of the MC/CL films were higher than those of the MC/chitosan films [11].

## 4. Conclusions

The introduction of CL impeded the aggregation and crystallization of MC molecular chains, suggesting the MC–CL molecular interactions. Only one glass transition temperature, which was between that of the MC and CL films, was observed for the MC/CL 7:3 and 5:5 films, respectively, indicating the good compatibility of these two blends, corresponding well with their relatively smooth morphologies and relatively good light transmittance properties. The MC/CL 5:5 film possessed both the higher rigidity of the MC film and the higher ductility of the CL film, representing its good flexibility. The hydrophobicity, water insolubility, and oxygen obstructing properties of the blended film increased with the increase in CL content. The MC/CL 5:5 film showed the lowest water vapor permeability. 

Overall, the MC/CL 5:5 film showed the best comprehensive properties, with improved hydrophobic, oxygen barrier, and water vapor barrier properties, as well as better elongation than the MC film, while also retaining the good light transmittance and tensile strength of the MC film. This research offers new MC based films with improved properties, exhibiting great potential for use as edible coatings, capsules, and packaging materials.

## Figures and Tables

**Table 1:** T_500_ of the MC/CL films with different blend ratios.

Sample	MC/CL 10:0	MC/CL 7:3	MC/CL 5:5	MC/CL 3:7	MC/CL 0:10
T_500_ (%)	83.68 ± 1.96 ^a^	79.05 ± 2.37 ^ab^	76.97 ± 5.44 ^b^	61.22 ± 3.17 ^c^	39.84 ± 4.04 ^d^

Note: Different letters indicate significant differences (*p* < 0.05).

## Data Availability

The datasets generated for this study are available on request to the corresponding author.
